# Composition, Properties, and Utilization of Fumaric Acid Sludge By-Produced from Industrial Phthalic Anhydride Wastewater Treatment

**DOI:** 10.3390/polym14235169

**Published:** 2022-11-28

**Authors:** Zhongjin Wei, Fengshan Zhou, Sinan Chen, Hongxing Zhao

**Affiliations:** 1 Beijing Key Laboratory of Materials Utilization of Nonmetallic Minerals and Solid Wastes, National Laboratory of Mineral Materials, School of Materials Science and Technology, China University of Geosciences (Beijing), Beijing 100083, China; 2 Fujian Jinhua Integrated Circuit Co., Ltd., Quanzhou 362261, China

**Keywords:** fumaric acid, maleic acid, fumaric acid sludge, filtrate loss reducer, phthalic anhydride, wastewater treatment

## Abstract

To understand fumaric acid sludge (FAS) systematically and comprehensively and find out how to utilize it, we conducted a series of characterization analyses on FAS. Fourier transform infrared (FT-IR) Spectra shows that the main component of FAS is fumaric acids and also contains a small amount of silicate. The nuclear magnetic resonance hydrogen (^1^H-NMR) spectrum also shows that fumaric acid accounted for a large proportion of FAS. The *X*-ray diffraction (XRD) shows that the main phase in FAS is fumaric acid, and there is also a small amount of Kaliophilite. After gas chromatography and mass spectrometry (GC-MS) and pyrolysis gas chromatography and mass spectrometry (Py-GC-MS) analysis, it indicates that the possible volatiles and pyrolysis products in FAS are fumaric acid, maleic acid, maleic anhydride, phthalic acid, etc. In the test of Liquid chromatography and mass spectrometry (LC-MS), we determined the contents of phthalic acid, fumaric acid, and maleic acid in FAS. The detailed mass content of each component in FAS is as follows: phthalic acid is about 0.10–0.15%; maleic anhydride is about 0.40–0.80%; maleic acid is about 18.40–19.0%; fumaric acid is about 55.00–56.90%; succinic anhydride is about 0.06–0.08%; acrylic acid is about 0.06–0.08%; malic acid is about 0.90–1.00%; acetic acid is about 0.10–0.20%; silicate is about 0.25–0.30%; phthalic anhydride is about 0.20–0.30%; water is about 24.30–24.80%. The filtrate loss reducer (PAAF) used in oilwell drilling fluids synthesized by FAS not only has excellent temperature and complex saline resistance, the API filtration loss (FL) was only 13.2 mL/30 min in the complex saline based mud, but is also cost-effective.

## 1. Introduction

**Figure 1:**
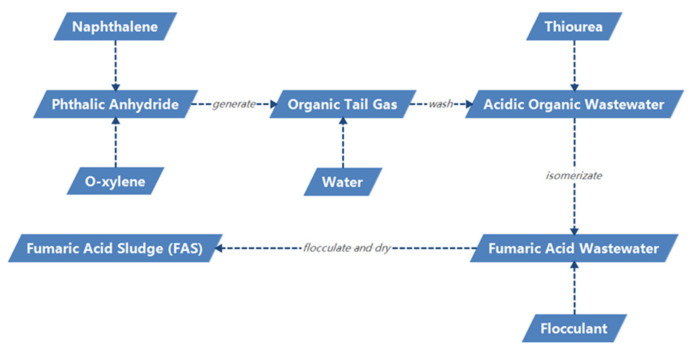
The generated process of fumaric acid sludge (FAS).

The organic tail gas generated in the production of phthalic anhydride will turn into acidic organic wastewater after washing with water [1,2]. Thiourea is added to acidic organic wastewater to make maleic acid isomerize into fumaric acid, and then after cooling, crystallization, and centrifugation, acidic organic wastewater becomes fumaric acid wastewater. The red and black fumaric acid wastewater was flocculated, precipitated, and dried to obtain powdery solid fumaric acid sludge (FAS). The generated process of fumaric acid sludge (FAS) is shown in Figure 1. A plant producing about 150,000 tons of phthalic anhydride will generate about 4000 tons of by-product FAS every year. FAS may contain hazardous chemicals such as naphthoquinone, naphthalene, maleic anhydride, etc., which will cause environmental pollution unless it is treated in a deep harmless manner [3,4]. With the increasing global demand for phthalic anhydride [5,6], a large amount of FAS will be generated, and it is necessary to find a simple and efficient method to deal with FAS.

The main method of FAS treatment is to separate and purify fumaric acid from FAS [7,8,9]. However, the separation and purification process are complex. Firstly, the anhydride wastewater was put into the acid tank for settlement; after settlement, activated carbon was added for decolorization, and then thiourea was added for transposition reaction, and the crude fumaric acid was obtained after filtration. Activated carbon and the crude fumaric acid solid were added to distilled water, heated to boiling to completely dissolve the crude fumaric acid solid, and heated reflux was added to filter out the activated carbon. After cooling to room temperature, the high-purity fumaric acid can be obtained after filtration and drying. The whole process has high requirements for equipment, high energy consumption, high treatment cost, and low economic benefit, so it is difficult to obtain high-purity fumaric acid [10,11,12]. Some researchers [13] have studied the use of FAS to produce polyol unsaturated polyester resin for the production of artificial marble, and foam resin for kitchen cabinets and home decoration building materials, but there is no industrial application. The specific composition of FAS varies with different synthetic process routes, operating process parameters of different manufacturers, and different organic wastewater treatment processes, etc. It is certain that FAS contains a large amount of fumaric acid and maleic acid, which can be widely used in the production of unsaturated resin, biopharmaceuticals, food additives, and other fields [14,15,16,17], so the high-value utilization of FAS is of great environmental and economic significance.

Drilling fluid is the “blood” of the oilfield drilling process, and the addition of various additives to drilling fluid can stabilize the well wall, balance the formation pressure, and carry broken rock cuttings, and so on [18]. With the continuous decline in oil prices, the development direction of drilling fluid additives is to use industrial by-products as raw materials to reduce the production cost of drilling fluid additives [19,20,21]. Filtrate loss reducer is the core additive of drilling fluid, which can reduce the amount of drilling fluid filtrate loss penetration into the formation interior to ensure safe oilfield production. In recent years, fumaric acid and maleic acid were used by many researchers to synthesize drilling fluid additives [22,23,24]. If FAS can be used as raw material to synthesize filtrate loss reducer for oil well drilling fluid, it will be a method to realize the high-value utilization of FAS.

In order to understand FAS systematically and comprehensively and find out the method to utilize FAS efficiently and with high value, in this work, firstly, we conducted a series of qualitative and quantitative analyses on FAS. Then, a method for synthesizing filtrate loss reducer with FAS as raw material is proposed, which can utilize FAS simply and efficiently. Finally, we evaluated the comprehensive performance of the filtrate loss reducer (PAAF), which was synthesized based on FAS. The PAAF has an excellent temperature and complex saline resistance, which is more cost-effective than similar products on the market.

## 2. Materials and Methods

### 2.1. Materials and Instrument

Acrylamide (AM) was purchased from Shandong Duofeng Chemical Co., Ltd., Shandong, China. 2-acrylamide-2-methylpropanesulfonic acid (AMPS) was purchased from Jingwen Dongxin Biotechnology Co., Ltd., Beijing, China. Potassium persulfate, sodium hydroxide, sodium carbonate, sodium bicarbonate, sodium chloride, anhydrous calcium chloride, magnesium chloride, fumaric acid, and maleic acid were purchased from Beijing Yili Fine Chemicals Co., Ltd., Beijing, China. Fumaric acid sludge (FAS) was obtained from Karamay Zhengcheng Co., Ltd., Karamay, China. Except for AM, AMPS, and FAS, which were industrial grade, the other reagents were analytical grade.

Roller heating furnace GRL-9, six-speed rotary viscometer ZNN-D6, digital display high-speed mixer GJ-2S, and multi-linked medium-pressure filtrate loss instrument ZNS-2A were manufactured in Qingdao Tongda Special Instrument Co., Ltd., Qingdao, China. Multi-functional high-speed blender SUS-304 was manufactured in Wuyi Hainan Electric Co., Ltd., Haikou, China. Electric thermostatic blast drying oven DHG-9035A was manufactured in Beijing Lushi Technology Co., Ltd., Beijing, China. Infrared spectrometer Nicolet IS5 was manufactured in Nicolet Corporation, Waltham, MA, USA. Full Digital NMR Spectrometer BRUKER-400MHz was manufactured in Bruker (Beijing) Technology Co., LTD., Beijing, China. *X*-ray Diffractometer XD-3 was manufactured in Beijing Pu-Analysis General Instrument Co., Ltd., Beijing, China. Gas chromatography-mass spectrometry GC-7890A/MS-5975c was manufactured in Agilent Technologies (China) Co., Ltd., Beijing, China. Pyrolysis gas chromatograph-mass spectrometer PY-3030D/QP2010Ultra was manufactured in Shimadzu (China) Co., Ltd., Beijing, China. Liquid chromatograph-mass spectrometer Acquity-TQD was manufactured in Waters Technology (Shanghai) Co., Ltd., Shanghai, China.

### 2.2. Analysis Method of FAS

#### 2.2.1. FT-IR

The samples were prepared using the KBr method. Firstly, the sample and KBr were ground into a fine powder in an agate mortar with a mass ratio of approximately 1:150. After being fully dried under an infrared lamp, the sample was pressed into a thin sheet using a tablet machine. Then, the thin sheet was carefully mounted on the magnetic sample rack and placed in the sample chamber of the Fourier infrared spectrometer for measurement. The prepared samples were scanned in a wave-number range of 4000 cm^−1^ to 400 cm^−1^ with a resolution of 4 cm^−1^ and a signal-to-noise ratio of 50,000:1. All spectrums were obtained by accumulating 64 scans.

#### 2.2.2. ^1^H-NMR

The FAS samples were fully dried in the oven and then dissolved with D2O and CD3OD as solvents, respectively. After 30 min of ultrasonic shaking at 40 °C, the samples were placed in a nuclear magnetic resonance hydrogen spectrometer and tested at a resonance frequency of 400 MHz. 

#### 2.2.3. XRD

The FAS samples were ground into a fine powder, compacted onto a test block to form a very flat surface, and then placed in the sample chamber of the X-ray diffractometer. The samples were tested under the parameters of a scan range of 2theta = 10–80°, step width = 0.01°, and a light tube power of 35 kV and 30 mA. 

#### 2.2.4. XRF

Take 3–5 g FAS and place it in the sample mold. Then, put the sample mold into the tablet machine and press it for 15 s at a pressure of 20 MPa. Determination of the elemental composition and its proportion in FAS was conducted using the *X*-ray fluorescence spectrometer. The analytical range of elements is Be (4)–U (92). 

#### 2.2.5. GC-MS

The FAS samples were pre-treated by the solid phase microextraction (SPME) method. The samples were determined by gas chromatography and mass spectrometry (GC-MS) and retrieved using the NIST mass spectrometry library. GC conditions: initial temperature of 50 °C, hold for 5 min, ramp up to 100 °C at 10 °C/min, then ramp up to 300 °C at 30 °C/min, hold for 4 min; the chromatographic column was DB-5(30 m × 0.25 mm × 0.25 μm). MS conditions: ion source was ESI; ion source temperature was 230 °C, maximum 250 °C; quadrupole temperature was 150 °C, maximum 200 °C; scan mass range was 29–500 AMU, ion source energy of 70 eV, emission current of 34.6 μA; run time of 10 min.

#### 2.2.6. Py-GC-MS

The FAS samples were placed in a pyrolysis reactor for pyrolysis, and the pyrolysis temperature increased from 50 °C to 550 °C at a rate of 10 °C/min. Volatile components pyrolysis from FAS samples were trapped in the cold trap at the port of the chromatographic column. Then, it enters GC-MS for separation and detection. The detection conditions are the same as those of Section 2.2.5.

#### 2.2.7. LC-MS 

The high-performance liquid chromatography (HPLC) system was used to separate the samples, and a mass spectrometry (MS) system was used for detection. LC conditions: the chromatographic column was Shimadzu XR-ODS 2.0 mmI.D. × 75 mmL, 2.2 μm; mobile phase: A is an aqueous solution; B is methanol; flow rate: 0.3 mL/min; column temperature: constant temperature 30 °C; elution mode: binary gradient elution; gradient elution procedure is shown in Table 1.

MS conditions: ion source: ESI; atomized gas: nitrogen at a flow rate of 3.0 L/min; DL temperature: 250 ℃; heating module temperature: 400 ℃; dry gas: nitrogen at a flow rate of 10.0 L/min. 

Preparation of standard solution: the standard solution is diluted in a gradient with water. Phthalate and malic acid are prepared as standard working solutions with concentrations of 0.15, 0.65, 1.30, 2.60, and 6.40 μg/mL; fumaric acid and sodium citrate are prepared as standard working solutions with concentrations of 0.60, 1.10, 2.20, 5.50, and 11.00 μg/mL; Sulfourea is prepared as a standard working solution with concentrations of 0.10, 0.50, 1.00, 2.00, and 5.00 μg/mL.

### 2.3. Preparation of PAAF Based on FAS

FAS pretreatment: FAS pretreatment: Block FAS was dried in an oven at 80 °C for 24 h and then crushed into a fine powder with a high-speed blender until ready to use.

Preparation method of the PAAF: Dissolve a certain mass of sodium hydroxide and sodium carbonate in deionized water, and then stir while adding a certain mass of FAS fine powder to obtain solution A; solution B was obtained by dissolving a certain mass of AMPS and AM in deionized water. Solution C was obtained by dissolving a certain mass of initiator K_2_S_2_O_8_ and cross-linking agent [25,26,27,28,29] Na_2_SiO_3_ in deionized water. The detailed composition of the solutions is shown in Table 2.

Transfer solution A and solution B to a three-mouth flask, mix well, and adjust pH to alkalinity. After 30 min of nitrogen injection into the three-mouth flask, the solution temperature was raised to 55 °C, and solution C was added drop by drop. After 5 h of reaction, the mixed solution turned into a brown viscous liquid, which was poured out and put into an oven at 80 °C to obtain the PAAF after drying.

### 2.4. PAAF Performance Evaluation Method

The performance evaluation of the PAAF is reflected by two parts of data: rheological parameters and Filtration loss FL_API_. The test methods are based on the test methods used by these researchers [30]. The specific test steps are as follows: 

Rheological parameters: Pour the drilling fluid mud sample into the sample cup of the rotational viscometer; make the liquid level reach the scale line in the sample cup of the rotational viscometer; put the sample cup on the bottom frame of the viscometer; move the bottom frame so that the sample liquid level coincides with the scale line on the outer cylinder; measure and record the temperature of the drilling fluid sample. Adjust the rotational speed of the outer cylinder of the rotational viscometer, and after the dial reading value is stabilized, read and record the dial reading value under different rotational speeds. Calculate the apparent viscosity, plastic viscosity, and dynamic shear force based on the following equation:

AV = R_600_/2 (1)

PV = R_600_ − R_300_ (2)

YP = 0.511(R_300_ − PV) (3)

where:

R_600_ = the reading of the viscometer at 600 r/min (dia);

R_300_ = the reading of the viscometer at 300 r/min (dia);

AV = apparent viscosity (mPa·s);

PV = plastic viscosity (mPa·s);

YP = yield point (Pa).

Filtration loss FL_API_: Pour the drilling fluid mud sample into the filtration loss meter cup; make the liquid level reach the scale line in the filtration loss meter cup, and put on the filtrate paper, and install the filtration loss meter; put the dry measuring cylinder underneath to receive the filtrate; close the pressure relief valve, and adjust the pressure regulator to make the pressure reach 690 ± 35 kPa (100 ± 5 psi) in 30 s or less; start timing while pressurizing; after reaching 30 min, measure the collected filtrate volume, which is the room temperature filtration loss FL_API_.

## 3. Results and Discussion

### 3.1. Analysis of FAS

#### 3.1.1. FI-IR

**Figure 2:**
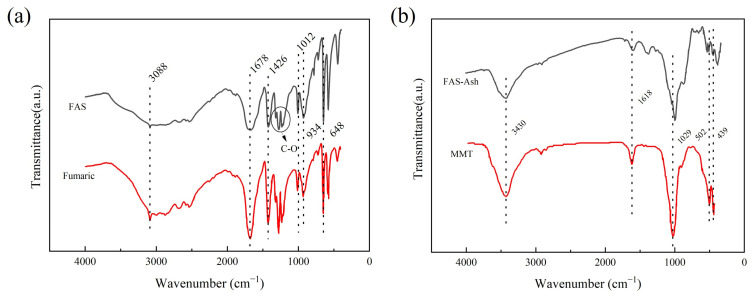
FT-IR spectrum comparison: (**a**) FAS and Fumaric; (**b**) FAS-Ash and MMT.

In the infrared spectrum of FAS (Figure 2), it shows an absorption peak at 3088 cm^−1^, which is caused by the =CH stretching vibration, while it shows the characteristic absorption peak at 1678 cm^−1^, which is caused by the C=O stretching vibration; the absorption peak occurs at 1426 cm^−1^, which is caused by the C-H in-plane bending vibration, and there are three peaks near 1276 cm^−1^, which are caused by the C-O stretching vibration. The characteristic peaks at 934 cm^−1^ and 648 cm^−1^ are caused by the out-of-plane bending vibration of O-H and the deformation vibration of O=C-O, respectively [31]. After searching and matching with the HR Aldrich FT-IR Collection Edition I standard spectrum library, it was found that the IR spectrum was highly consistent with that of the fumaric acid standard sample, indicating that the main component in FAS is fumaric acid. In the IR spectrum of the FAS-Ash, the hydroxyl stretching vibration peak and hydroxyl bending vibration peak in Al(Mg)OH occur at 3430 cm^−1^ and 1618 cm^−1^, respectively; 1029 cm^−1^, 502 cm^−1^, and 439 cm^−1^ are the characteristic peaks caused by the Si-O-Si skeleton vibration, O-Si-O asymmetric bending vibration, and O-Si-O symmetric bending vibration, respectively [32], and after searching and matching, the positions of the main absorption peaks of the FAS-Ash and Montmorillonite (MMT) standard sample IR profiles were consistent, indicating that the main component of the FAS-Ash is montmorillonite, and the FAS also contains a small amount of silicate.

#### 3.1.2. ^1^H-NMR

**Figure 3:**
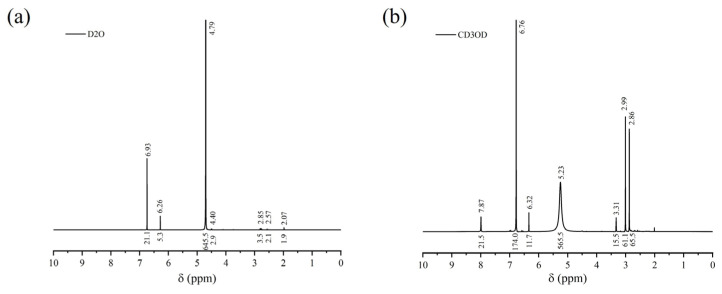
^1^H-NMR spectrum of FAS: (**a**) D2O; (**b**) CD3OD.

The NMR hydrogen spectra of FAS are shown in Figure 3. Figure 3a shows the hydrogen spectrum of FAS obtained by dissolving with D2O; Figure 3b shows the hydrogen spectrum of FAS obtained by dissolving with CD3OD. In Figure 3a, the peaks appear near δ = 6.93 ppm, 6.26 ppm, 4.79 ppm, 4.40 ppm, 2.85 ppm, 2.57 ppm, 2.07 ppm, where the peak at δ = 4.79 ppm is the residual solvent peak. The peaks appearing at δ = 6.93 ppm and 6.26 ppm are the chemical shifts of CH(2,5) and CH(3,4) in pyrrole; the peak near δ = 4.40 ppm is the chemical shift of CH_3_ in nitromethane; the peak near δ = 2.85 ppm is the chemical shift of CH_3_ in dimethylformamide; the peak near δ = 2.57 ppm is the chemical shift of CH_2_ in triethylamine; the peak near δ = 2.07 ppm is the chemical shift of CH_3_CO in ethyl acetate [33]. In Figure 3b, the peaks appear at δ = 7.87 ppm, 6.76 ppm, 6.32 ppm, 5.23 ppm, 3.31 ppm, 2.99 ppm, 2.86 ppm, where the peak at δ = 3.31 ppm is the residual solvent peak; the peak at δ = 7.87 ppm may be the chemical shift of CH in trichloromethane; the peak at δ = 6.76 ppm may be the chemical shift of CH(2,5) in pyrrole; the peak at δ = 5.23 ppm is the chemical shift of HC = CH in fumaric acid (or maleic acid); the peaks at δ = 2.99 ppm and 2.86 ppm are the chemical shift of CH_3_ in dimethylformamide [33]. The residual solvent peak in the hydrogen spectrum obtained by dissolving FAS with D2O is relatively broad and long, and its area is large (the area of the residual solvent peak is 645.5, and the sum of the remaining peak areas is about 36.8), which indicates that only a small portion of FAS can be dissolved in D2O, thus making the residual solvent peak the main peak of the hydrogen spectrum. The residual solvent peak (δ = 3.31 ppm) in the NMR hydrogen spectrum obtained by dissolving FAS with CD3OD is relatively narrow and short, while the peak at δ = 5.23 ppm (the chemical shift of HC=CH in fumaric acid) appears wider and has a larger area (the peak area at δ = 5.23 ppm is 565.5; the residual solvent peak area is 15.5; and the total area of the remaining peaks is about 333.8), which indicates that FAS is basically soluble in CD3OD, and the percentage of fumaric acid (or maleic acid) in FAS accounted for a larger proportion, which was consistent with the FT-IR characterization results.

#### 3.1.3. XRD

**Figure 4:**
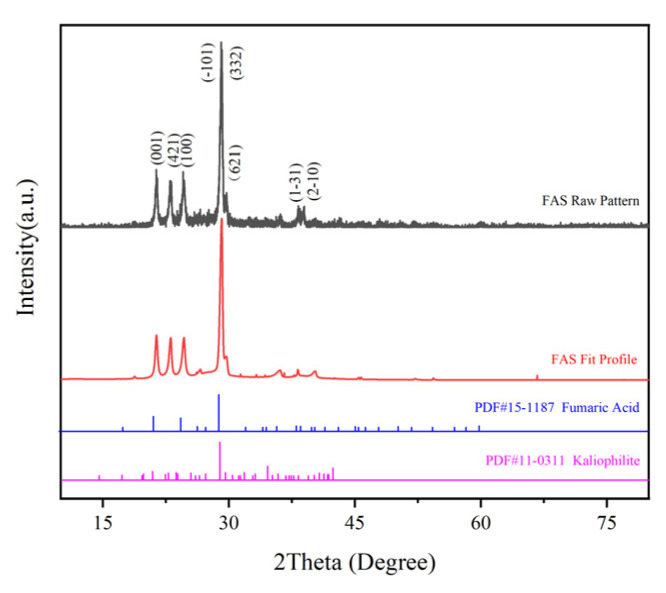
XRD spectrum of FAS.

The XRD spectrum of FAS is shown in Figure 4. The main peak positions of this spectrum correspond to the main peaks of Fumaric Acid (PDF#15-1187) and Kaliophilite (PDF#11-0311); the diffraction peaks at 2Theta = 21.1°, 24.4°, 28.9°, 38.2°, and 38.7° correspond to the (001), (100), (-101), (1-31), and (2-10) crystal planes of Fumaric Acid, respectively, and the diffraction peaks at 2Theta = 22.7°, 28.9°, and 29.6° correspond to the (421), (332), and (621) crystal planes of Kaliophilite, respectively. This indicates that the main phase in FAS is fumaric acid, and a small amount of Kaliophilite (silicate minerals) is also present, which is consistent with the analysis results of the FT-IR spectrum of FAS and FAS-Ash. In addition, there are more burr spurious peaks in the spectrum, which indicates that the crystalline phase of FAS is not pure; FAS contains a lot of heterogeneous substances, which are less but more diverse.

#### 3.1.4. XRF

The XRF test results of FAS are shown in Table 3. From Table 3, it can be seen that the FAS samples mainly contain carbon, calcium, silicon, phosphorus, sodium, aluminum, magnesium, and other elements, indicating that FAS does contain silicate substances, among which silicon, sodium, aluminum, magnesium, and calcium are from silicate substances. From the elemental ratio, the proportion of carbon is as high as 99.91%, and the proportion of total elements other than carbon is less than 0.1%, which indicates that the proportion of silicate substances in FAS is very small, and FAS is mainly composed of organic substances.

#### 3.1.5. GC-MS and Py-GC-MS

**Figure 5:**
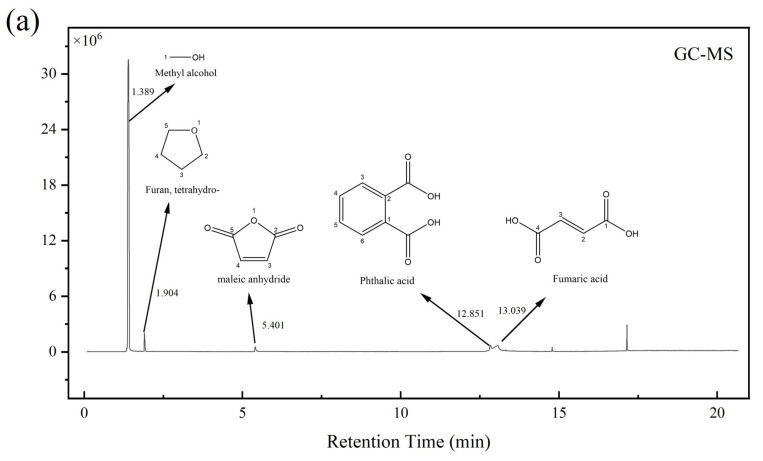

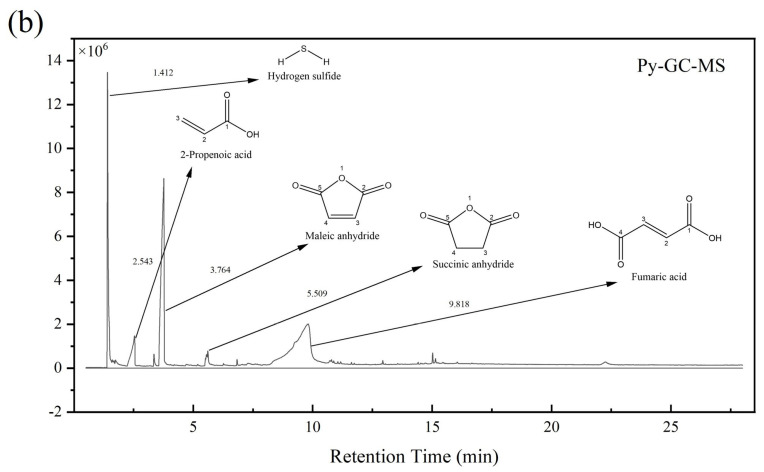
Chromatograms of FAS: (**a**) GC-MS; (**b**) Py-GC-MS.

The chromatograms of FAS are shown in Figure 5, (a) GC-MS, (b) Py-GC-MS. Both chromatograms show many wave peaks, indicating that there are many volatiles and pyrolysis products in FAS. In Figure 5a, the main peak positions RT = 1.389, 1.904, 5.401, 12.851, 13.039 min correspond to the volatile products of Methyl alcohol, Furan, Maleic anhydride, Phthalic acid, and Fumaric acid. In Figure 5b, the main peak positions RT = 1.412, 2.543, 3.764, 5.509, and 9.818 min correspond to the cleavage products Hydrogen sulfide, 2-propenoic acid, Maleic anhydride, Succinic anhydride, Fumaric acid, respectively. After matching with the NIST mass spectrometry standards database, the possible volatiles and pyrolysis products in FAS were obtained, and the detailed list is shown in Table 4 and Table 5.

After reviewing the relevant documents and combining the products retrieved from the database, the volatiles in FAS were analyzed as fumaric acid, maleic acid, maleic anhydride, phthalic acid, etc. The high-temperature pyrolysis products were fumaric acid, maleic anhydride, acetic acid, acrylic acid, succinic anhydride, phthalic anhydride, etc. The trace high-temperature pyrolysis products were quantified using the external standard method (ultrasound as the pretreatment method and ME as the dilution solvent). The results of the quantitative analysis are shown in Table 6.

Table 5 shows that the high-temperature pyrolysis products of FAS contain about 0.146% acetic acid, 0.067% acrylic acid, 0.070% succinic anhydride, and 0.239% phthalic anhydride, and the total amount of these components does not exceed 1%. This indicates that the content of volatile substances other than fumaric acid and maleic acid in FAS is low, and the total amount of fumaric acid and maleic acid may account for more than 99% of FAS if water is not considered.

#### 3.1.6. LC-MS

**Figure 6:**
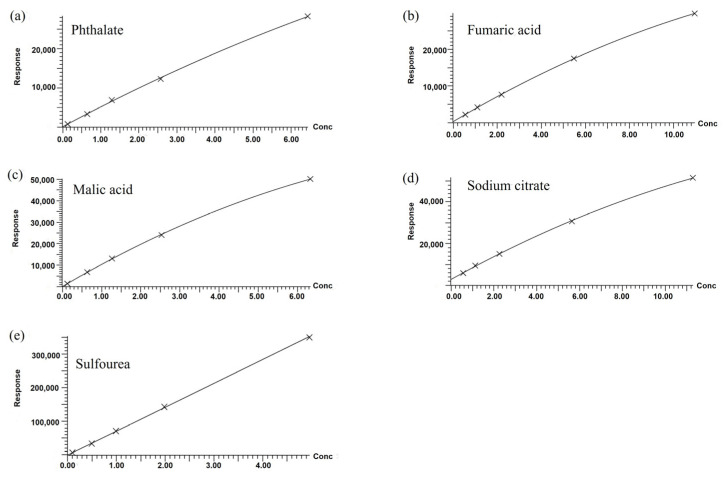
The standard working curve for water-soluble components in FAS: (**a**) Phthalate; (**b**) Fumaric acid; (**c**) Malic acid; (**d**) Sodium citrate; (**e**) Sulfourea.

**Figure 7:**
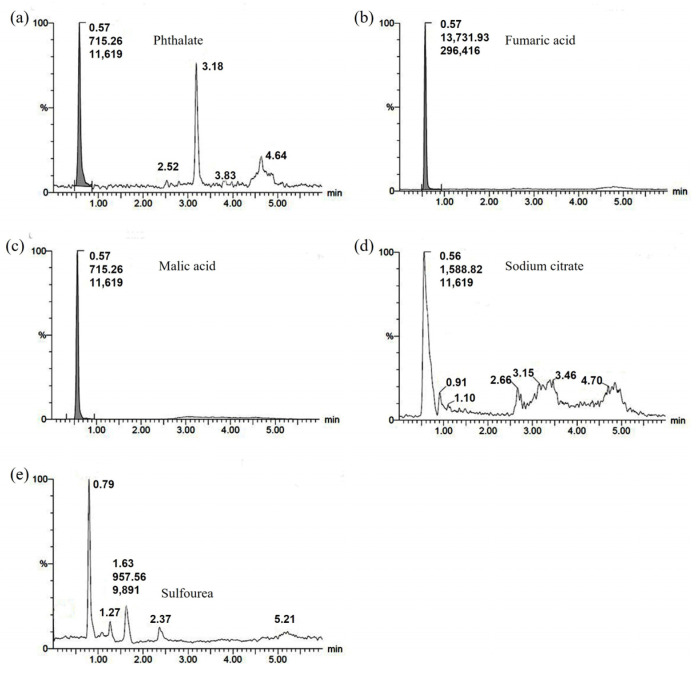
Chromatograms for water-soluble components in FAS: (**a**) Phthalate; (**b**) Fumaric acid; (**c**) Malic acid; (**d**) Sodium citrate; (**e**) Sulfourea.

Based on the analysis of FAS testing, certain water-soluble components that may be present in FAS were quantified, which include Sulfourea, sodium citrate, phthalic acid, malic acid, and fumaric acid. The standard working curve was first produced by using the external standard method with these five components as the targets, as shown in Figure 6, and then the chromatograms of each component were obtained by using the water fixation and dilution method, as shown in Figure 7. The instrument detected 0.116 μg/mL of phthalic acid, 4.149 μg/mL of fumaric acid, 0.957 μg/mL of malic acid, and no sulfourea and sodium citrate (the detection limit of the instrument is 0.1 μg/mL). Based on the volume of water fixed volume converted into a content percentage, the content of phthalic acid is about 0.11%; malic acid is about 0.93%; and fumaric acid is about 40.48% (probably the total amount of fumaric acid and maleic acid, during the test, as well as the fumaric acid in the sample, was not dissolved completely, resulting in a low quantitative result of fumaric acid).

Generally speaking, it is difficult to obtain the exact mass content of each component in the sample through a single characterization. Therefore, we combined the results of multiple characterization analyses, such as LC-MS, XRD, XRF, etc., and combined with our research group’s experience in waste analysis over the years, to infer the mass content range of each component in FAS: phthalic acid is about 0.10–0.15%; maleic anhydride is about 0.40–0.80%; maleic acid is about 18.40–19.0%; fumaric acid is about 55.00–56.90%; succinic anhydride is about 0.06–0.08%; acrylic acid is about 0.06–0.08%; malic acid is about 0.90–1.00%; acetic acid is about 0.10–0.20%; silicate is about 0.25–0.30%; phthalic anhydride is about 0.20–0.30%; water is about 24.30–24.80%.

### 3.2. Performance Evaluation of PAAF

Some common commercial filtrate loss reducers (PAS, SN, and FRS) were selected to compare performance with the PAAF. PAS is a polymer filtrate loss reducer with a polycarboxylic acid structure, which can effectively reduce the viscosity of drilling mud; SN is sodium polyacrylonitrile, which has excellent resistance to NaCl; FRS is a filtrate reducer suitable for offshore oil and gas wells, with good sea salt resistance.

To study the effect of FAS on the AM/AMPS/FAS copolymerization system, we synthesized two kinds of PAAF, PAAF110 (without FAS) and PAAF111 (mass ratio AM:AMPS: FAS = 1:1:1). Weigh two portions of a certain mass of the sample and add them to two groups of complex saline based mud, respectively. One portion was stirred at a high speed for 20 min, and then stirred at a high speed for 10 min after 24 h of maintenance to determine the room temperature filtration loss FL_API_ and rheological parameters; the other group was stirred at high speed for 20 min, loaded into a high-temperature aging tank, hot rolled and aged at 150 °C for 16 h; after it cools, take out and stir at a high speed for 10 min, and then determine the room temperature filtration loss FL_API_ and rheological parameters.

#### 3.2.1. Rheological Performance of PAAF

**Figure 8:**
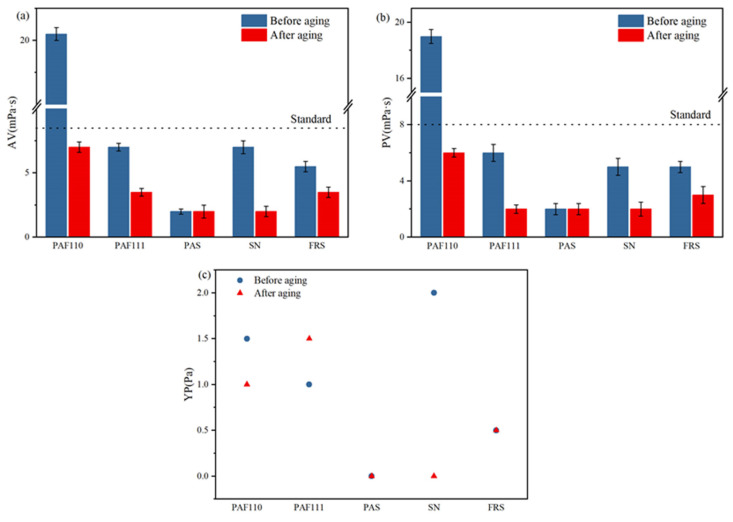
The rheological parameters of all samples before and after aging: (**a**) AV; (**b**) PV; (**c**) YP.

It can be seen from Figure 8 that after 16 h of hot rolling and aging at 150 °C, the AV and PV of the mud all decreased, indicating that the molecular structure of all samples was damaged to a certain extent at a high temperature of 150 °C. From the YP point of view (Figure 8c), the YP value of the mud with SN is quite different before and after aging, before aging YP = 2.0 Pa, and after aging YP = 0.0 Pa, indicating that the molecular structure of SN is damaged worse at a high temperature. The rheological parameters (AV, PV, and YP) of PAAF111 before and after hot rolling and aging are not significantly different from other commercial filtrate loss reducers (PAS, SN, and PRS), while the AV and PV values of PAAF110 before aging are higher, AV = 20.5 mPa·s, PV = 19.0 mPa·s, all of which are higher than the industry standard of filtrate loss reducers (≤8 mPa·s). If only AM and AMPS are used to synthesize the polymer (PAAF110), it will have a high degree of polymerization and high molecular weight. After adding PAAF110 into the drilling mud, the viscosity of the mud will be greatly increased. After a certain quality of FAS is added to the AM/AMPS copolymerization system, a large number of COOH groups are introduced into the molecular structure of the synthesized polymer (PAAF111), and the presence of COOH can reduce the degree of polymerization and the molecular weight of the polymer. After it is added to the drilling mud, it will not increase the viscosity of the mud too much. In terms of rheological performance, the synthesis of PAAF111 based on FAS (mass ratio AM:AMPS: FAS = 1:1:1) can meet the industry standard of filtrate loss reducers used in oil wells, which is at the same level as other commercial filtrate loss reducers.

#### 3.2.2. Temperature and Complex Saline Resistance Performance of PAAF

**Figure 9:**
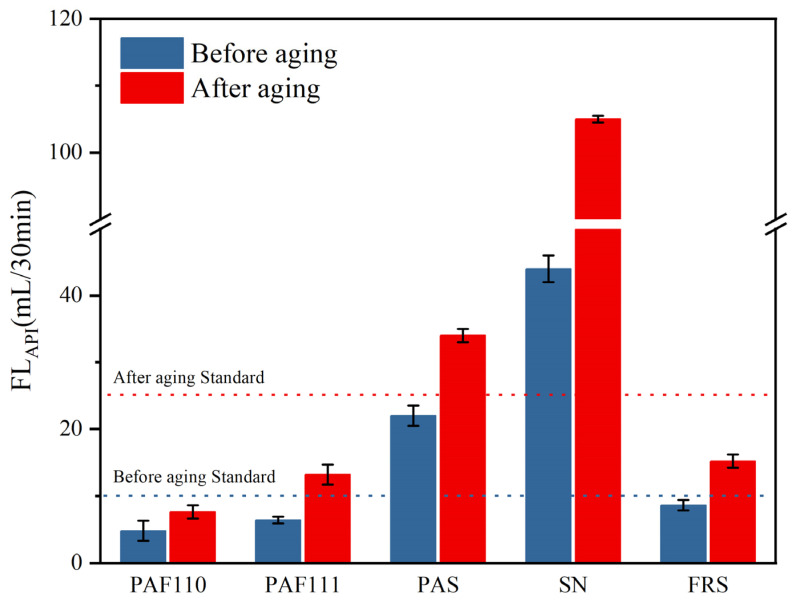
The FL_API_ of all samples before and after aging.

Figure 9 shows the FL_API_ of all samples before and after hot rolling and aging. Before aging, the FL_API_ of PAS and SN exceeded the industry standard, indicating that the structure of PAS and SN was damaged in the complex saline based slurry. After aging, the FL_API_ of PAS and SN also exceeded the industry standard, which showed that the temperature resistance of PAS and SN was poor. The FL_API_ of SN after hot rolling and aging at 150 °C is as high as 105.0 mL/30 min, indicating that its molecular structure is destroyed at a high temperature and loses the function of reducing filtration. Among PAAF110, PAAF111, and FRS, the FL_API_ of PAAF110 before and after hot rolling and aging is the smallest, while the FL_API_ of PAAF111 before and after hot rolling and aging is slightly lower than that of FRS, which means that with the addition of FAS, although the temperature and complex saline resistance performance of the PAAF is slightly decreased, it is still better than the commercial filtrate loss reducer (FRS) on the market. The FL_API_ of PAAF111 in complex saline based mud before hot rolling and aging is only 6.4 mL/30 min, indicating that it has excellent complex saline resistance, and the FL_API_ after 150 °C hot rolling and aging for 16 h is only 13.2 mL/30 min, indicating that its main molecular structure is not damaged at a high temperature, and its temperature resistance is excellent. In terms of temperature and complex saline resistance performance, the PAAF is better than common commercial filtrate loss reducer products, and it is a product with excellent performance.

#### 3.2.3. The Cost-Effective of PAAF

After a series of performance comparisons, we found that after the FAS participates in AM/AMPS copolymerization, it can not only effectively improve the rheological performance of drilling mud (before aging, AV is reduced from 20.5 mPa·s to 7.0 mPa·s), but also the loss of temperature and complex saline resistance performance is not large (after aging, FL_API_ increased from 7.6 mL to 13.2 mL). In terms of production cost, the cost of PAAF110 obtained by copolymerization of AM and AMPS is more than 18,000 CNY/t, and the PAAF111 copolymerized after adding a certain quality of FAS can greatly reduce the production cost under the condition that the performance decline is small; the cost can be controlled at about 12,000 CNY/t. Therefore, using FAS to prepare PAAF can not only make use of FAS but also make the product with price advantages.

For comparison, the market price of PAAF and some commercial filtrate loss reducers (PAS, SN, FRS) are shown in Table 7. It can be seen that compared with PAS and SN, the filtration loss FL_API_ of the PAAF in the complex saline based mud is lower. Compared with FRS, the PAAF has a similar performance but a lower price. The PAAF prepared based on FAS will have a very optimistic market prospect in the field of the oil well drilling fluid filtrate loss reducer.

## 4. Conclusions

After a series of qualitative and quantitative analyses of FAS, the detailed mass content of each component in FAS was obtained, as follows: phthalic acid is about 0.10–0.15%; maleic anhydride is about 0.40–0.80%; maleic acid is about 18.40–19.0%; fumaric acid is about 55.00–56.90%; succinic anhydride is about 0.06–0.08%; acrylic acid is about 0.06–0.08%; malic acid is about 0.90–1.00%; acetic acid is about 0.10–0.20%; silicate is about 0.25–0.30%; phthalic anhydride is about 0.20–0.30%; water is about 24.30–24.80%.

We proposed an industrialized utilization method of FAS: copolymerized with AM/AMPS to synthesize a filtrate loss reducer for oilfield drilling. There is no refining requirement for FAS, and it can be used directly for polymerization after a simple drying process, a simple method to achieve efficient utilization of waste FAS.

The oil well drilling fluid filtrate loss reducer PAAF synthesized based on FAS not only has a better temperature and complex saline resistance, FL_API_ in complex saline mud is 6.4 mL before hot rolling and aging and 13.2 mL after hot rolling and aging at 150 °C for 16 h, but also has a price advantage compared to the common commercial filtrate loss reducers.

## Figures and Tables

**Table 1:** Gradient elution procedure.

Time (min)	A (Water)	B (Methanol)
1.00	90	10
2.00	60	40
4.00	10	90
6.00	10	90
6.01	90	10

**Table 2:** Composition of solutions.

Solution A	Solution B	Solution C
sodium hydroxide NaOH	acrylamide AM	potassium persulfate K_2_S_2_O_8_
sodium carbonate Na_2_CO_3_	2-acrylamide-2-methyl propane sulfonic acid AMPS	sodium silicate Na_2_SiO_3_
fumaric acid sludge FAS		

**Table 3:** Elemental composition of FAS.

Chemical Element	Conc. (%)
C (carbon)	99.911
Ca (calcium)	0.0468
Si (silicon)	0.0165
P (phosphorus)	0.0095
Na (sodium)	0.0078
Al (aluminum)	0.0059
Mg (magnesium)	0.0027

**Table 4:** Volatiles in FAS retrieved by computer.

Retention Time/min	Compounds	Molecular Formula	CAS No.
1.389	Methyl alcohol	CH_4_O	67-56-1
1.497	Acetone	C_3_H_6_O	67-64-1
1.611	2-Propenoic acid	C_3_H_4_O_2_	79-10-7
1.740	Acetic acid	C_2_H_4_O_2_	64-19-7
1.757	2-Butanone	C_4_H_8_O	78-93-3
1.875	Trichloromethane	CHCl_3_	67-66-3
1.904	Furan	C_4_H_8_O	109-99-9
2.286	2,3-Butanedione	C_4_H_6_O_2_	431-03-8
5.401	Maleic anhydride	C_4_H_2_O_3_	108-31-6
9.236	Decane	C_10_H_22_	124-18-5
12.851	Phthalic anhydride	C_8_H_4_O_3_	85-44-9
13.039	Fumaric acid	C_4_H_4_O_4_	110-17-8
13.316	4-tert-Amylphenol	C_11_H_16_O	80-46-6
15.535	n-Hexadecanoic acid	C_16_H_32_O_2_	57-10-3
16.1802	Octadecanoic acid	C_18_H_36_O_2_	57-11-4

**Table 5:** Pyrolysis products retrieved by computer.

Retention Time/min	Compounds	Molecular Formula	CAS No.
1.412	Hydrogen sulfide	H_2_S	7783-06-4
1.461	1,3-Butadiene	C_4_H_6_	106-99-0
1.603	Hydrogen chloride	HCl	7647-01-0
1.737	Acetic acid	C_2_H_4_O_2_	64-19-7
2.543	2-Propenoic acid	C_3_H_4_O_2_	79-10-7
2.721	Toluene	C_7_H_8_	108-88-3
3.528	Methacrylic acid	C_4_H_6_O_2_	79-41-4
3.662	Ethylbenzene	C_8_H_10_	100-41-4
3.764	Maleic anhydride	C_4_H_2_O_3_	108-31-6
3.786	p-Xylene	C_8_H_10_	106-42-3
4.043	Styrene	C_8_H_8_	100-42-5
4.049	1,3-dimethyl	C_8_H_10_	108-38-3
5.176	1,2,4-trimethyl	C_9_H_12_	95-63-6
5.509	Succinic anhydride	C_4_H_4_O_3_	108-30-5
5.745	Indene	C_9_H_8_	95-13-6
5.846	Phenol, 2-methoxy	C_7_H_8_O_2_	90-05-1
5.985	Acetophenone	C_8_H_8_O	98-86-2
6.778	3-methyl-1H-Indene	C_10_H_10_	767-60-2
7.977	1-Indanone	C_9_H_8_O	83-33-0
8.096	1-Methylnaphthalene	C_11_H_10_	90-12-0
8.344	Phthalic anhydride	C_8_H_4_O_3_	85-44-9
9.818	Fumaric acid	C_4_H_4_O_4_	110-17-8
10.247	Fluorene	C_13_H_10_	86-73-7

**Table 6:** Quantitative analysis of high-temperature pyrolysis products.

Sample	Mass/g	The Volume of Fixation/mL	Substances to be Measured	Instrument Reading Value/(mg/L)	Content/%	Average Content/%
1	1.0046	10	Acetic acid	148.42	0.148	0.146
			Acrylic acid	65.56	0.065	0.067
			Succinic anhydride	68.77	0.068	0.070
			Phthalic anhydride	235.13	0.234	0.239
2	1.0071	10	Acetic acid	146.24	0.145	-
			Acrylic acid	70.06	0.070	
			Succinic anhydride	71.67	0.071	
			Phthalic anhydride	245.96	0.244	

**Table 7:** Market price and performance comparison of the fluid loss reducer products.

Samples	Market Price (CNY/t)	AV (mPa·s)		FL_API_((mL/30 min))	
		Before Aging	After Aging	Before Aging	After Aging
Standards	-	≤8.0	≤8.0	≤10	≤25
PAAF110	18,000	20.5	8.0	4.8	7.6
PAAF111	12,000	7.0	3.5	6.4	13.2
PAS	8000	2.0	2.0	22.0	34.0
SN	12,000	7.0	2.0	44.0	105.0
FRS	15,000	5.5	3.5	8.6	15.2

## Data Availability

Data are available from the authors upon request.
